# 48-week prognostic analysis of very low-level viremia in patients with hepatitis B cirrhosis: a single-center retrospective study

**DOI:** 10.3389/fmed.2025.1549791

**Published:** 2025-07-02

**Authors:** Yinong Feng, Zehong Wang, Shaoyuan Shi, Li Zhou, Yongli Hua, Xuanxuan Wang, Jianzhong Ma

**Affiliations:** ^1^Department of Hepatology, The Third People’s Hospital of Taiyuan, Taiyuan, China; ^2^Science and Education Department, The Third People’s Hospital of Taiyuan, Taiyuan, China

**Keywords:** chronic hepatitis B, cirrhosis, hepatocellular carcinoma, high sensitivity of HBV DNA detection, very low-level viremia

## Abstract

**Objective:**

Despite improvements in the accuracy of hepatitis B virus (HBV) DNA detection, some patients with chronic hepatitis B (CHB) still have very low-level viremia (VLLV; HBV DNA is detectable but less than 20 IU/mL) after achieving a complete virologic response (CVR). This study aimed to investigate the prognosis of patients with cirrhotic hepatitis B and VLLV.

**Methods:**

A total of 267 patients with hepatitis B cirrhosis from the Third People’s Hospital of Taiyuan were retrospectively enrolled. All patients took oral antiviral drugs for more than 96 weeks and were divided into the target not-detected group (TND; HBV DNA undetectable) and the VLLV group (limits of detection < HBV DNA < 20 IU/mL) by high-sensitivity testing of HBV. The incidence of cirrhosis-related complications was observed.

**Results:**

Compared to the TND group, the baseline levels of alanine aminotransferase (ALT; 20.0 vs. 26.0 U/L, *p* < 0.001), aspartate aminotransferase (AST; 24.0 vs. 27.5 U/L, *p* < 0.001), and gamma-glutamyl transferase (GGT; 21.0 vs. 30.5 U/L, *p* = 0.001) were significantly higher in the VLLV group, and so were liver stiffness values (9.4 vs. 10.8 kPa, *p* = 0.006). No significant difference was observed in the rate of new cirrhosis-related complications between the two groups. The HCC rate was 5.4% in TND and 4.7% in the VLLV (*p* > 0.05). Multifactorial logistic regression showed that the main factors affecting complications at baseline were age (OR:1.063; *p* = 0.034), hemoglobin level (OR:0.965; *p* = 0.036), and platelet count (OR:0.987; *p* = 0.029).

**Conclusion:**

For cirrhotic patients with VLLV, the lower the level of HBV DNA, the less severe the liver injury. There was no difference in the 48-week complication rates between the TND group. Even in the TND group, which can develop new complications, regular follow-up should be performed.

## Introduction

Chronic hepatitis B virus (HBV) infection is a common infectious disease that jeopardizes human health. Approximately 25% of patients with cirrhosis and 30% of patients with hepatocellular carcinoma are directly infected with HBV (1, 2). In 2019, approximately 820,000 individuals died of HBV-related liver failure, cirrhosis, and hepatocellular carcinoma (HCC) (3, 4). Although the past four decades have witnessed a significant decline in HBV infection in China, approximately 43.3 million persons remained infected with HBV in 2021 (5). About 15–40% of people with untreated chronic HBV infection progress to liver cirrhosis or HCC (6). Therefore, the early diagnosis and treatment of these patients are essential.

The basic principle in the treatment of chronic hepatitis B (CHB) is to maximize the long-term inhibition of HBV replication, thereby alleviating hepatitis and liver fibrosis, minimizing complications, and improving patient prognosis (7). Currently, the first-line antiviral drugs in major domestic and international guidelines are divided into two main categories: nucleoside/nucleotide analogs (NAs), such as Entecavir (ETV), Tenofovir disoproxil fumarate (TDF), and Tenofovir alafenamide (TAF), and pegylated interferon-alpha (8–11). HBV DNA level is an important indicator of the effectiveness of antiviral therapy; however, some patients cannot achieve complete virologic response (CVR) or maintain virologic response and even develop low-level viremia (LLV, detectable HBV DNA but less than 2000 IU/mL), partly because of their high baseline HBV DNA level and poor treatment compliance during long-term treatment. Recent studies have suggested that persistent LLV is significantly associated with poor clinical outcomes, which may be a cause of HCC in patients receiving NA treatment (12). Thus, CHB patients with LLV imply that the current antiviral therapy is less effective and that the antiviral regimen needs to be adjusted if necessary.

Controlling HBV DNA levels below the lower limit of detection, clinically considered as the CVR (most studies defined as 20 IU/mL), effectively reduced the incidence of cirrhosis and HCC (13–15). Notably, with the widespread application of highly sensitive HBV DNA testing, it has been found that CHB patients achieving CVR still have very low-level viremia (VLLV, detectable HBV DNA but less than 20 IU/mL). However, studies on their clinical prognosis are limited (16). Some studies have suggested that the lower the level of HBV DNA, the lower the probability of developing HCC in CHB patients with LLV (17). However, others were convinced that there was no significant difference in the survival rate between patients with HBV DNA levels less than 10 IU/mL and those with HBV DNA levels between 10 and 20 IU/mL (14). Therefore, we conducted a retrospective study to explore whether the long-term presence of VLLV affects the prognosis of patients with cirrhotic hepatitis B.

## Methods

### Patient selection

**Figure 1:**
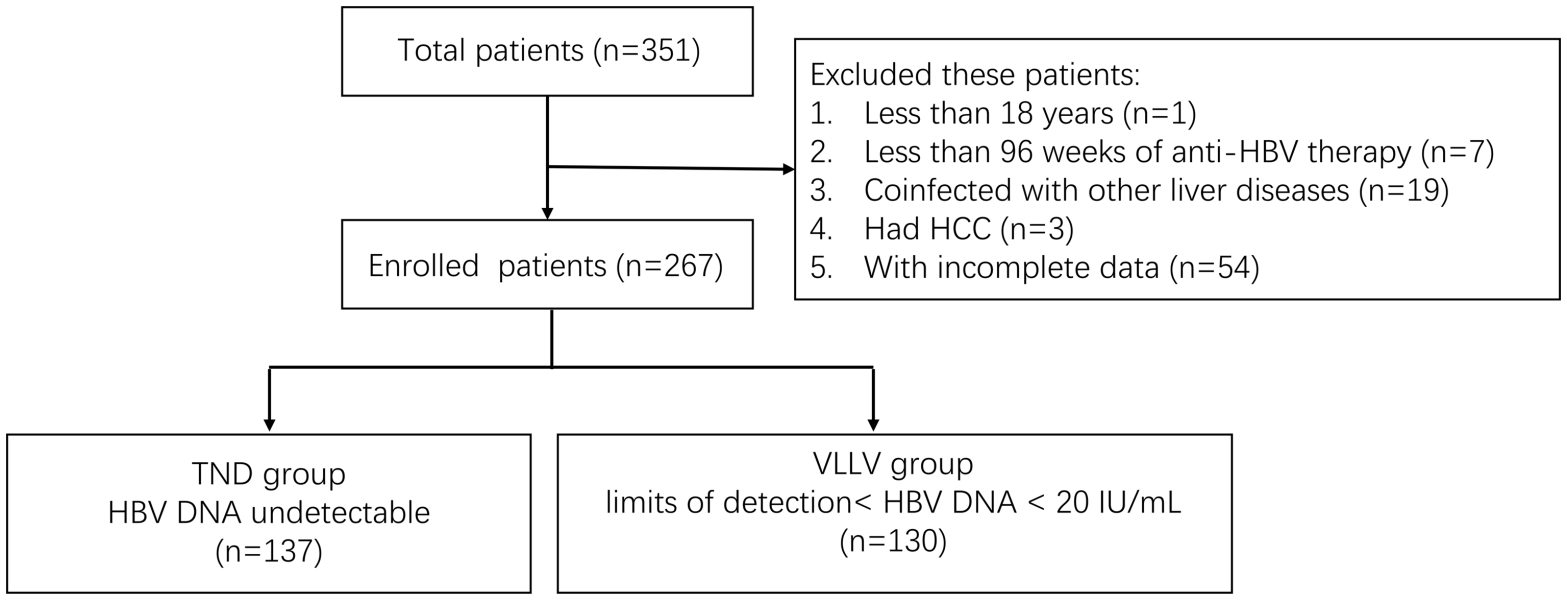
Flow chart for the selection of study participants. TND: target not detected; VLLV: very low-level viremia; HCC: hepatocellular carcinoma; HBV: hepatitis B virus.

We collected the following data from the patients: (1) age>18 years; (2) cirrhotic hepatitis B diagnostic criteria of the Chinese guidelines (2019 edition) (18); (3) oral antiviral drugs for at least 96 weeks; and excluded patients who (1) were co-infected with hepatitis A virus, hepatitis C virus, hepatitis D virus, hepatitis E virus, human immunodeficiency virus, Epstein–Barr virus, or cytomegalovirus; (2) patients with other liver diseases, such as alcohol-related liver disease, drug-induced liver disease, or autoimmune liver disease; and (3) had HCC. A total of 267 patients treated for cirrhotic hepatitis B were included from the Third People’s Hospital of Taiyuan from September 1, 2020, to December 31, 2022. All patients achieved CVR clinically, i.e., HBV DNA < 20 IU/mL. They were divided into the TND group (HBV DNA undetectable, *n* = 137) and the VLLV group (limits of detection < HBV DNA < 20 IU/mL, *n* = 130) based on the baseline results of high-sensitivity HBV DNA detection (Figure 1). The study protocol was approved by the Ethics Committee of Taiyuan No.3 Hospital (no. 2020-06) and was conducted in compliance with the Declaration of Helsinki. The requirement for informed consent was waived by the Ethics Committee.

### Measurements

Blood biochemical and virological parameters were collected at baseline and 48 weeks. Each patient underwent high-sensitivity testing for hepatitis B virus (hepatitis B virus nucleic acid detection kit, PCR-fluorescence method; Roche Diagnostic Products Co. Ltd.). Biochemical indices included alanine aminotransferase (ALT), aspartate aminotransferase (AST), alkaline phosphatase (ALP), gamma-glutamyl transferase (GGT), total bilirubin (TB), direct bilirubin (DB), albumin (ALB), creatinine (CR), blood lipids, and prothrombin time (PT). Liver stiffness measurement (LSM) and controlled attenuation parameter (CAP) were examined using transient elastography (FibroTouch, HISKY, CHINA). The main complications of concern during the follow-up period were abdominal effusion, hypersplenism, esophageal and gastric varices, hepatic encephalopathy, portal vein thrombosis, and primary HCC.

### Outcomes

The primary outcome was the cumulative rate of HBV-related complications during the follow-up period. The secondary outcome was alterations in liver function, including ALT, AST, ALP, and GGT levels, before and after the follow-up.

### Calculation of sample size

In this study, the patients were divided into the VLLV and TND groups, and the primary outcome was the proportion of cirrhotic patients who developed cirrhosis-related complications during antiviral therapy. A parallel two-group design was used to test whether the variance of the VLLV group was equivalent to that of the TND group by testing whether the variance ratio was between 0.5 and 2. Taking the incidence of Type I error as 0.05 and Type II error as 0.15 and the ratio of VLLV and TND groups as 1:1, the sample size of each group was calculated as 82 using PASS software (version 25.0.2). At a dropout rate of 15%, 97 patients per group were required, resulting in a final requirement of 194 patients.

### Statistical analysis

Statistical analyses were performed using SPSS (Statistical Package for the Social Sciences, IBM Corp., United States) and the R language. Quantitative variables are expressed as median (interquartile range, IQR) and compared using the Wilcoxon rank-sum test. Categorical variables were compared using the chi-squared test or Fisher’s exact test. All statistical tests were two-tailed, and *a* value< 0.05 was considered statistically significant. Variables with a *p* value< 0.10 in the univariable logistic regression model were included in the multivariate model. Factors predicting 1-year new complications were identified using a multivariate logistic regression analysis.

## Results

### Baseline liver injury indicators were higher in the VLLV group than in the TND group

There were no statistically significant differences between the VLLV and TND groups in terms of age, height, weight, alcohol consumption, family history of hepatitis B, proportion of complications, or presence of decompensated cirrhosis (Table 1). Clinical examination results showed that the levels of ALT (20.0 vs. 26.0 U/L, *p* < 0.001), AST (24.0 vs. 27.5 U/L, *p* < 0.001), GGT (21.0 vs. 30.5 U/L, *p* = 0.001), and alpha fetal protein (2.23 vs. 2.78 ng/mL, *p* = 0.010) were significantly higher in the VLLV group compared with the TND group (Table 2). Notably, the percentage of patients with ALT or AST > 40 U/L in the VLLV group exceeded 20%, compared with less than 10% in the TND group. In addition, FibroTouch testing also indicated that the LSM values were higher in the VLLV group (9.4 vs. 10.8 kPa, *p* = 0.006). These results suggest that very low levels of replicating HBV DNA contribute to persistent hepatic injuries in patients with cirrhotic hepatitis B who had achieved CVR.

**Table 1:** Baseline demographic data in the TND group and the VLLV group.

Physical examination items	TND (*n* = 137)	VLLV (*n* = 130)	*p*-value
Age (year) (137:130)	55.0 (47.0–61.0)	51.0 (42.0–59.0)	**0.003**
Male (%) (137:130)	80 (58.4%)	86 (66.2%)	0.377
Height (cm) (137:130)	168.0 (160.0 ~ 172.0)	169.5 (163.0 ~ 172.0)	0.132
Weight (kg) (137:130)	66.0 (60.0–73.0)	70.0 (60.6–75.0)	0.080
Body mass index (kg/m^2^) (137:130)	24.1 (22.3–26.3)	24.2 (22.4–26.7)	0.278
Overweight (>23.0)	88 (64.7%)	90 (69.2%)	0.681
Drinking history (136:130)	20 (14.7%)	24 (18.5%)	0.638
Family history of HBV (136:130)	69 (50.7%)	80 (61.5%)	0.130
Hypertension (137:130)	12 (8.8%)	10 (7.7%)	0.526
Hyperglycemia (137:130)	8 (5.8%)	6 (4.6%)	0.188
Complication (137:130)	86 (62.8%)	72 (55.4%)	0.426
Ascites	7 (5.1%)	5 (3.8%)	
Varicosity	82 (59.9%)	70 (53.8%)	
Hypersplenism	46 (33.6%)	43 (33.1%)	
Decompensated LC (137:130)	42 (30.7%)	37 (28.5%)	0.673

Continuous variables were expressed as median and interquartile range (IQR). Categorical variables are presented as frequencies (percentages). The number in parentheses in the first column represents the number of individuals included in the statistics. The chi-squared test or Fisher’s exact test was used for categorical variables. The Kruskal–Wallis test was used to analyze continuous variables. LC: liver cirrhosis; TND: target not detected (HBV DNA < limits of detection); VLLV: very low level viremia (limits of detection ≤HBV DNA <20 IU/mL). Bold text indicated that the differences were statistically significant.

**Table 2:** Baseline laboratory data in the TND group and the VLLV group.

Testing items	TND (*n* = 137)	VLLV (*n* = 130)	*p-*value
Hepatitis B e antigen positive (135:130)	18 (13.3%)	51 (39.2%)	<0.001
MELD score (137:130)	4.54 (2.39–6.31)	3.86 (2.56–6.29)	0.568
Child–Pugh level≥B (137:130)	11 (8.0%)	11 (8.5%)	0.320
Blood routine			
White blood cell (G/L) (134:127)	4.60 (3.40–5.50)	4.40 (3.30–5.20)	0.522
Red blood cell (G/L) (133:125)	4.42 (4.11–4.82)	4.70 (4.07–4.97)	0.065
Hemoglobin (g/L) (133:125)	144 (129–156)	146 (131–160)	0.139
Platelet (G/L) (134:127)	157.00 (104.75–205.75)	134 (97.00–187.50)	0.108
Neutrophile granulocyte (G/L) (133:125)	2.42 (1.85–3.10)	2.50 (1.81–3.06)	0.862
Lymphocyte (G/L) (133:125)	1.45 (1.05–2.04)	1.48 (1.21–1.82)	0.910
Blood chemistry			
Total bilirubin (μmol/L) (137:130)	17.26 (13.00–21.94)	15.42 (12.57–23.58)	0.492
Direct bilirubin (μmol/L) (137:130)	3.59 (2.59–4.59)	3.21 (2.47–4.87)	0.501
Alanine aminotransferase (U/L) (137:130)	20.0 (14.0–28.0)	26.0 (19.0–42.8)	**<0.001**
≥40 U/L (%)	10 (7.3%)	36 (27.7%)	
Aspartate aminotransferase (U/L) (137:130)	24.0 (20.0–29.0)	27.5 (22.0–35.0)	**<0.001**
≥40 U/L (%)	8 (5.8%)	26 (20.0%)	
Alkaline phosphatase (U/L) (114:112)	90.0 (69.5–110.0)	89.0 (76.8–112.5)	0.347
Gamma-glutamyl transferase (U/L) (115:112)	21.0 (15.0–34.0)	30.5 (18.8–50.0)	**0.001**
Albumin (g/L) (137:130)	43.0 (39.0–46.0)	43.0 (40.0–46.0)	0.693
Globulin (g/L) (137:129)	29.0 (27.0–33.0)	30.0 (27.0–32.0)	0.836
Creatinine (μmol/L) (137:130)	64.0 (52.0–69.0)	62.5 (55.0–70.8)	0.531
Uric acid (μmol/L) (122:119)	285.5 (240.0–338.0)	293.0 (242.5–361.0)	0.210
Fasting blood glucose (mmol/L) (118:108)	5.40 (4.94–5.91)	5.48 (4.93–6.08)	0.446
Total cholesterol (mmol/L) (113:111)	3.94 (3.37–4.61)	4.17 (3.51–4.70)	0.311
Triglyceride (mmol/L) (113:111)	1.02 (0.74–1.37)	0.96 (0.75–1.31)	0.907
High-density lipoprotein (mmol/L) (113:109)	1.07 (0.96–1.26)	1.14 (0.93–1.34)	0.364
Low-density lipoprotein (mmol/L) (113:109)	2.15 (1.75–2.53)	2.22 (1.80–2.61)	0.429
Blood phosphorus (mmol/L) (106:94)	1.05 (0.94–1.22)	1.10 (0.94–1.19)	0.839
Alpha fetoprotein (ng/mL) (128:123)	2.23 (1.66–3.60)	2.78 (1.95–4.14)	**0.010**
Prothrombin time (s) (137:130)	11.9 (11.3–12.7)	11.8 (11.20–12.6)	0.591
Activated partial thromboplastin time (s) (137:130)	32.8 (30.6–34.5)	33.2 (31.4–35.4)	0.090
International normalized ratio (137:130)	1.12 (1.06–1.21)	1.12 (1.06–1.21)	0.802
Other indicators			
Liver stiffness measurement (kPa) (137:130)	9.4 (7.0–11.6)	10.8 (7.7–15.5)	**0.006**
Controlled attenuation parameter (dB/m) (137:130)	245 (225–267)	241 (221–264)	0.428

Continuous variables were expressed with median and interquartile range (IQR). Categorical variables are presented as frequencies (percentages). The number of parentheses in the first column represents the number of individuals included in the statistics. The chi-squared test or Fisher’s exact test was used for categorical variables. The Kruskal–Wallis test was used for continuous variables. TND: target not detected (HBV DNA < limits of detection); VLLV: very low-level viremia (limits of detection ≤HBV DNA <20 IU/mL). Bold text indicated that the differences were statistically significant.

### No significant difference was observed in the 1-year incidence of cirrhosis-related complications between the two groups

**Figure 2:**
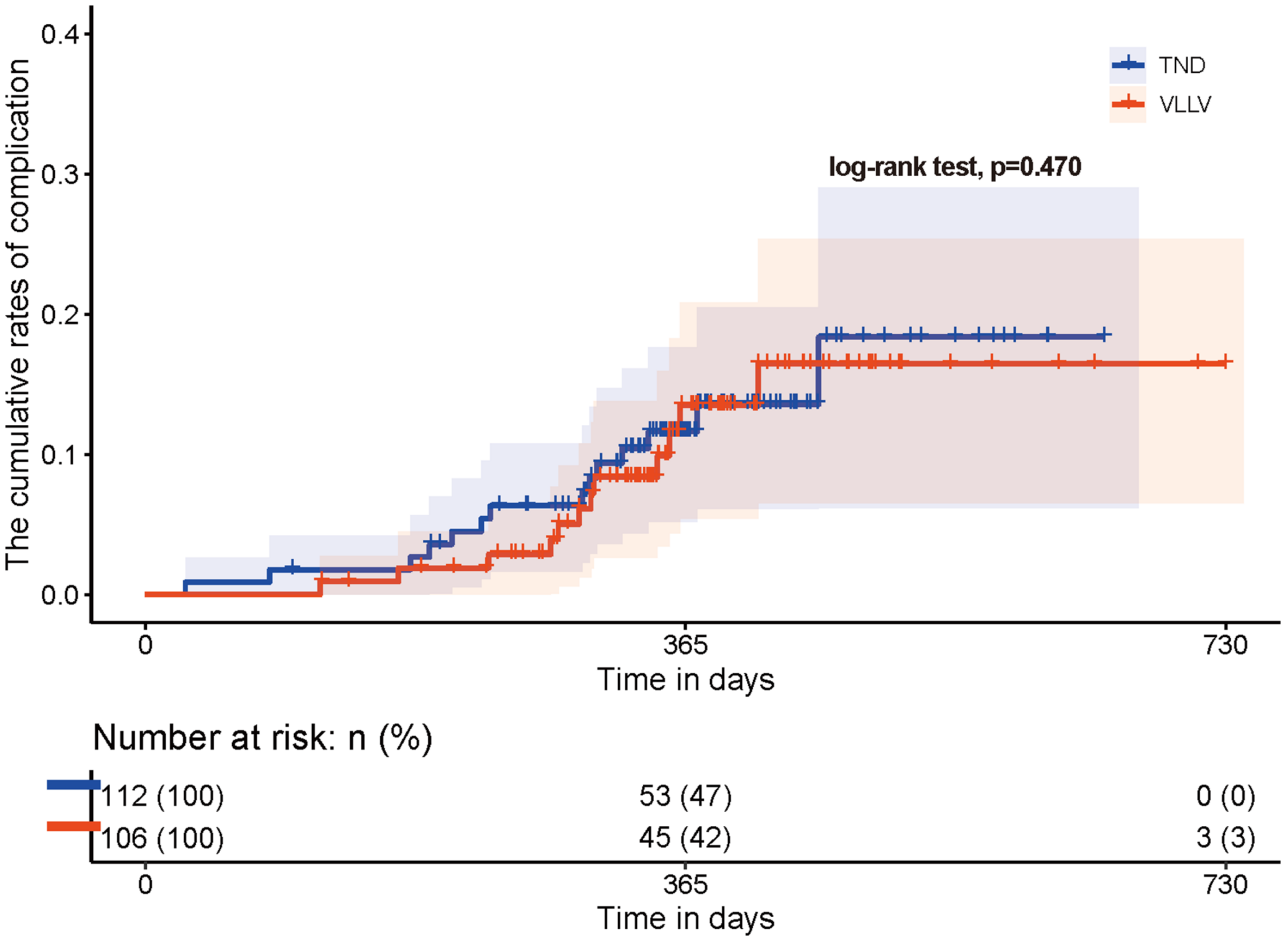
Cumulative incidence of HBV-related complications. There was no significant difference in the cumulative incidence of complications between the TND and VLLV groups at almost 730 days of follow-up (using the log-rank test; *p* = 0.470). TND: target not detected; VLLV: very low-level viremia.

New HBV-related complications occurred during the follow-up period; however, there was no statistically significant difference in the cumulative rates between the VLLV and TND groups (Figure 2, log-rank test, *p* > 0.05). The types of complications are shown in Table 3. There was no significant difference in the incidence of HCC between groups. A total of 6 patients in the TND group (5.4%) and 5 patients in the VLLV group (4.7%) developed HCC, and the median time to diagnosis of HCC was 233 days and 303 days, respectively. In conclusion, for patients with cirrhotic hepatitis B whose HBV DNA is less than 20 IU/mL, there was no direct correlation between HBV DNA levels and the development of complications. However, it should be noted that cirrhotic patients with undetectable HBV DNA can develop new complications.

**Table 3:** New complications in the TND group and the VLLV group during 48-week follow-up.

Types of complications	TND (*n* = 112)	VLLV (*n* = 106)	*p* value
Hypersplenism	0 (0.0%)	2 (1.9%)	0.235
Esophageal and gastric varices	0 (0.0%)	2 (1.9%)	0.235
Ascites	7 (6.3%)	5 (4.7%)	0.769
Upper gastrointestinal hemorrhage	6 (5.4%)	0 (0.0%)	0.029
Portal vein thrombosis	5 (4.5%)	0 (0.0%)	0.060
HCC	6 (5.4%)	5 (4.7%)	0.829
Total	16 (14.3%)	13 (12.3%)	0.660

Categorical variables are presented as frequencies (percentages). The chi-squared test or Fisher’s exact test was used for categorical variables. HCC: hepatocellular carcinoma, TND: target not detected (HBV DNA < limits of detection), VLLV: very low level viremia (limits of detection ≤HBV DNA <20 IU/mL).

### The liver injury indices were still higher in the VLLV group than in the TND group after one-year follow-up

The blood biochemical parameters and FibroTouch results were compared before and after follow-up. After a median follow-up of 361 (329–416) days, 112 patients were followed up in the TND group and 106 patients in the VLLV group. Compared with baseline, the levels of ALT, AST, GGT, PLT, ALB, and LSM changed insignificantly in both groups (Table 4). However, after 48 weeks of antiviral treatment, the levels of ALT (22.0 vs. 20.0 U/L, *p* = 0.011), GGT (28.5 vs. 23.0 U/L, *p* = 0.050), and LSM (10.5 vs. 8.7, *p* = 0.018) in the VLLV group were still higher than those in the TND group.

**Table 4:** The changes of clinical indices in the TND group and the VLLV group during 48-week follow-up.

Testing items		TND (*n* = 112)			VLLV (*n* = 106)		
	Baseline	48-week	*p*-value^1^	Baseline	48-week	*p*-value^1^	*p*-value^2^
ALT (U/L) >40 U/L (%)	21.0 (15.0–28.8) 7 (6.3%)	20.0 (15.0–26.0) 11 (9.8%)	0.828	25.0 (19.0–39.3) 25 (23.6%)	22.0 (19.0–31.8) 17 (16.0%)	0.099	**0.011**
AST (U/L) >40 U/L (%)	23.0 (20.0–30.0) 8 (7.1%)	23.0 (19.0–29.0) 10 (8.9%)	0.613	26.0 (22.0–33.3) 12 (11.3%)	26.0 (20.0–31.0) 8 (7.5%)	0.062	0.165
GGT (U/L)	20.0 (15.0–35.0)	23.0 (16.0–36.5)	0.052	31.0 (18.0–49.0)	28.5 (17.8–42.5)	0.445	**0.050**
PLT (G/L)	157.0 (101.0–210.0)	164.5 (112.5–200.5)	0.845	131.0 (96.0–186.8)	140.5 (94.0–183.2)	0.602	0.061
ALB (g/L)	43.0 (39.0–46.0)	43.0 (40.0–46.0)	0.994	43.0 (41.0–46.0)	44.0 (40.8–47.0)	0.130	0.144
LSM (kPa)	9.3 (6.8–11.3)	8.7 (7.0–11.9)	0.730	11.0 (7.6–15.8)	10.5 (7.2–14.7)	0.781	**0.018**

Continuous variables were expressed with median and interquartile range (IQR). Categorical variables are presented as frequencies (percentages). *p*-value^1^ represented the use of a paired-sample test within each group, and *p*-value^2^ represented the use of an independent-sample test between the TND and VLLV groups at 48 weeks. ALT: alanine aminotransferase, AST: aspartate aminotransferase; ALB: albumin; GGT: gamma-glutamyl transferase; LSM: liver stiffness measurement; TND: target not detected (HBV DNA < limits of detection); VLLV: very low level viremia (limits of detection ≤HBV DN A < 20 IU/mL). Bold text indicated that the differences were statistically significant.

### Factors predicting complications

Univariate logistic regression analysis was performed to identify meaningful factors. The results showed that age, decompensated liver cirrhosis, PT, LSM, MELD score, and Child–Pugh score were significantly positively associated with the occurrence of complications, whereas WBC, RBC, HGB, PLT, and ALB were negatively correlated. These variables were included in a multivariate logistic regression model (Table 5). The results showed that age (OR 1.063; 95% CI: 1.005–1.125), hemoglobin level (OR: 0.965; 95% CI: 0.933–0.998), and platelet count (OR 0.987; 95% CI: 0.975–0.999) were independent predictive factors for complications.

**Table 5:** Predictive factors of HBV-related complications.

Variable	Univariable analysis		Multivariate analysis	
	OR (95% CI)	*p*-value	OR (95% CI)	*p*-value
Gender	0.757 (0.344–1.666)	0.490		
Age	1.043 (1.003–1.086)	0.037	1.063 (1.005–1.125)	0.034
BMI	0.997 (0.884–1.124)	0.955		
Hypertension	1.338 (0.362–4.942)	0.662		
Hyperglycemia	1.481 (0.304–7.226)	0.627		
Family history of HBV	1.377 (0.607–3.122)	0.444		
Drinking history	0.979 (0.580–1.653)	0.938		
Decompensated LC	6.451 (2.791–14.911)	<0.001	1.016 (0.283–3.651)	0.981
Complication	5.562 (1.864–16.601)	0.002	1.235 (0.277–5.502)	0.782
WBC	0.612 (0.450–0.832)	0.002	1.230 (0.814–1.860)	0.325
RBC	0.391 (0.198–0.771)	0.007	3.095 (0.711–13.466)	0.132
HB	0.973 (0.957–0.989)	0.001	0.965 (0.933–0.998)	0.036
PLT	0.981 (0.972–0.989)	<0.001	0.987 (0.975–0.999)	0.029
TB	1.024 (0.999–1.048)	0.055		
ALT	1.002 (0.981–1.024)	0.824		
AST	1.000 (0.993–1.007)	0.925		
ALP	1.008 (0.997–1.019)	0.176		
GGT	1.011 (0.994–1.029)	0.206		
ALB	0.870 (0.806–0.940)	<0.001	0.960 (0.851–1.082)	0.501
AFP	1.016 (0.950–1.086)	0.643		
PT	2.166 (1.597–2.937)	<0.001	1.432 (0.920–2.229)	0.112
CR	0.973 (0.941–1.006)	0.112		
HBsAg	1.000 (1.000–1.000)	0.340		
HBeAg	0.789 (0.542–1.148)	0.215		
LSM	1.101 (1.017–1.192)	0.018	1.015 (0.906–1.137)	0.802
CAP	0.995 (0.984–1.006)	0.340		
MELD score	1.217 (1.069–1.387)	0.003	1.018 (0.826–1.253)	0.870
Child–Pugh scores	2.365 (1.597–3.5043)	<0.001	1.382 (0.847–2.255)	0.196

AFP: alpha fetoprotein; ALT: alanine aminotransferase; AST: aspartate aminotransferase; ALB: albumin; ALP: alkaline phosphatase; CAP: controlled attenuation parameter; CHB: chronic hepatitis B; CR: creatinine; GGT: gamma-glutamyl transferase; HB: hemoglobin; HBV: hepatitis B virus; HCC: hepatocellular carcinoma; IQR: interquartile range; LC: liver cirrhosis; LSM: liver stiffness measurement; PLT: platelet; PT: prothrombin time; RBC: red blood cell; TB: total bilirubin; WBC: white blood cell.

## Discussion

Highly sensitive HBV DNA testing of patients with cirrhotic hepatitis B who achieved CVR revealed that some still had very low levels of HBV DNA (lower limit of detection, ~20 IU/mL). Whether this affects patient prognosis remains to be investigated. In our study, we followed up with this group of patients for 48 weeks and preliminarily answered this question. The patients were divided into the VLLV and TND groups based on their HBV DNA levels at baseline. Comparison of results before and after follow-up showed that antiviral therapy did not significantly improve the values of ALT, AST, GGT, PLT, ALB, and LSM in either group, indicating that liver function in cirrhotic patients who achieved CVR was relatively stable and that continued antiviral therapy was able to maintain this status. However, the between-group comparison showed that the values of ALT, GGT, and LSM were higher in the VLLV group than in the TND group, both at baseline and at 48 weeks of follow-up, suggesting that very low levels of HBV replication may have a long-term potential impact on the improvement of hepatitis and liver cirrhosis. Theoretically, higher liver enzymes may indicate more severe liver injury, often suggesting poor prognosis (19, 20). Patients who achieved CVR but continued to have elevated ALT levels were less likely to have reverse cirrhosis (21). In this study, there was no significant difference in the incidence of complications, including HCC, between the VLLV and TND groups. We suspect that this result may be related to the fact that this study did not include a long follow-up period. Therefore, whether the differences in liver enzyme and LSM levels are clinically meaningful requires further long-term studies.

The main predicted variables of complications were age, hemoglobin level, and platelet count (Table 5), suggesting that more attention should be paid to the regular follow-up of cirrhotic patients with advanced age but low hemoglobin levels and platelets. In theory, the possibility of adverse events should be reduced when the virus is negative in patients with hepatitis B. Many researchers have also used HBV DNA, ALT, and AST as prognostic markers in patients with chronic HBV infection (22, 23). However, in cirrhotic patients with HBV DNA < 20 IU/mL, ALT (OR: 1.002; 95% CI: 0.981–1.024; *p* = 0.824), AST (OR: 1.000; 95% CI: 0.993–1.007, *p* = 0.925), and HBV DNA appeared to have limited predictive power for complications. Obviously, long-term antiviral therapy can normalize the above indicators; however, clinicians should not let their guard down in these populations, and their complication rates are not low. Currently, the main indicator of antiviral efficacy is HBV DNA level, but there is a lack of further monitoring indices of antiviral efficacy when the patients’ HBV DNA is below the lower limit of detection. Recently, some studies have indicated that drug efficacy can be further judged based on the results of HBV RNA after HBV DNA turns negative (24–26). Furthermore, HBV DNA combined with RNA showed superiority in reflecting intrahepatic cccDNA activity in treatment-naïve CHB patients (27). However, since HBV RNA is not routinely tested in the clinic, a model based on routine blood indicators can indirectly determine the disease conditions of cirrhotic patients with hepatitis B who have achieved CVR.

Recently, it has also been proposed to further lower the standard of LLV. Both US and European guidelines set HBV DNA less than 10 IU/mL as the criterion for the limit of detection of HBV DNA (8, 9), whereas the Asia-Pacific guidelines specify a virological response level of 12 IU/mL (28). However, it is noteworthy that the majority of clinical studies set the CVR criterion at 20 IU/mL, which may be related to commercial test kits. It has been suggested that differences in the level of definition of CVR may have a significant impact on the progression of liver fibrosis and HCC in patients with CHB (16). Another point of concern was the prognosis in the TND group. Theoretically, the development of HCC can be decreased by achieving and maintaining CVR in patients with CHB. However, a recent study showed that the 5-year incidence of HCC in patients with hepatitis B cirrhosis who achieved CVR (HBV DNA < 12 IU/mL) was approximately 10% (17). In conclusion, the prognosis of patients with hepatitis B cirrhosis with undetectable HBV DNA is not optimistic regardless of the CVR criteria. Therefore, we should not relax the management of patients with hepatitis B cirrhosis. In other words, regular reexamination is necessary even in patients with undetectable viruses. There is also a need to search for new and easy-to-detect markers to make a scientific judgment of disease progression between VLLV and TND groups. However, the significance of the definition of CVR in patients with non-cirrhotic or cirrhotic CHB receiving long-term medication needs to be further evaluated.

Taken together, our results suggest that VLLV has an adverse impact on liver function and liver stiffness in cirrhotic patients but has a limited effect on 48-week HBV-related complications when compared to the TND group.

This retrospective study has several limitations. For example, because of the follow-up time of only one year in this study, we are not sure whether VLLV affects long-term prognosis in cirrhotic patients with hepatitis B. Second, the prediction model of liver cirrhosis complications needs to be verified with more clinical data. Large-scale, multicenter, prospective cohort studies are required to validate the findings of this study. Moreover, whether the type of NAs affects antiviral effectiveness needs further exploration.

## Data Availability

The original contributions presented in the study are included in the article/supplementary material, further inquiries can be directed to the corresponding authors.

## Glossary

AFP: alpha-fetoprotein
ALT: alanine aminotransferase
AST: aspartate aminotransferase
ALB: albumin
ALP: alkaline phosphatase
CAP: controlled attenuation parameter
CHB: chronic hepatitis B
CVR: complete virologic response
CR: creatinine
DB: direct bilirubin
ETV: Entecavir
GGT: gamma-glutamyl transferase
HB: hemoglobin
HBV: hepatitis B virus
HCC: hepatocellular carcinoma
IQR: interquartile range
LC: liver cirrhosis
LLV: low-level viremia
LSM: liver stiffness measurement
PLT: platelet
PT: prothrombin time
RBC: red blood cell
VLLV: very low-level viremia,
TAF: Tenofovir alafenamide
TB: total bilirubin
TDF: Tenofovir disoproxil fumarate
TND: target not detected,
WBC: white blood cell
